# Protein Interaction Network Reconstruction Through Ensemble Deep Learning With Attention Mechanism

**DOI:** 10.3389/fbioe.2020.00390

**Published:** 2020-05-05

**Authors:** Feifei Li, Fei Zhu, Xinghong Ling, Quan Liu

**Affiliations:** ^1^School of Computer Science and Technology, Soochow University, Suzhou, China; ^2^Provincial Key Laboratory for Computer Information Processing Technology, Soochow University, Suzhou, China

**Keywords:** protein-protein interaction network, protein-protein interaction, ensemble learning, deep learning, attention mechanism, multi-layer convolutional neural network

## Abstract

Protein interactions play an essential role in studying living systems and life phenomena. A considerable amount of literature has been published on analyzing and predicting protein interactions, such as support vector machine method, homology-based method and similarity-based method, each has its pros and cons. Most existing methods for predicting protein interactions require prior domain knowledge, making it difficult to effectively extract protein features. Single method is dissatisfactory in predicting protein interactions, declaring the need for a comprehensive method that combines the advantages of various methods. On this basis, a deep ensemble learning method called EnAmDNN (Ensemble Deep Neural Networks with Attention Mechanism) is proposed to predict protein interactions which is an appropriate candidate for comprehensive learning, combining multiple models, and considering the advantages of various methods. Particularly, it encode protein sequences by the local descriptor, auto covariance, conjoint triad, pseudo amino acid composition and combine the vector representation of each protein in the protein interaction network. Then it takes advantage of the multi-layer convolutional neural networks to automatically extract protein features and construct an attention mechanism to analyze deep-seated relationships between proteins. We set up four different structures of deep learning models. In the ensemble learning model, second layer data sets are generated with five-fold cross validation from basic learners, then predict the protein interaction network by combining 16 models. Results on five independent PPI data sets demonstrate that EnAmDNN achieves superior prediction performance than other comparing methods.

## Introduction

Protein interactions and interaction networks take part in vital activities of each living cell, including signal transduction, immune response, metabolism of energy substance, cell cycle control, etc. (Keskin et al., 2016). The exact identification of protein interactions is therefore important not only to understanding the functions of proteins but also to structure-based drug design and treatment of diseases (Li et al., 2009).

Majority of existing methods for predicting PPI are based on Gene Ontology and annotations, phylogenetic profile, gene fusion, the interacting proteins co-evolution pattern and the similarity of proteins in sequence, structure, domain and subcellular localization (Boxem et al., 2008; Zhang et al., 2012; Planas-Iglesias et al., 2013; Sun et al., 2017). However, as their accuracy and reliability depend heavily on collected prior knowledge, they are hardly applied widely. Several methods based on amino acid sequence computation have been explored to predict PPI, such as support vector machine with traditional auto-correlation, k-nearest neighbor (kNN) with local description(LD) (Yang et al., 2010), support vector machine (SVM) with conventional auto covariance(AC) (Guo et al., 2008) or local description(LD) (Zhou et al., 2011), deep neural network with amphiphilic Pseudo amino acid composition (PseAAC) descriptor (Du et al., 2017b) and so on. The above methods provide different techniques of protein sequences such as AC, LD, MCD, PseAAC, with each technique extracting different feature information of protein interactions (Zhang et al., 2019a). AC and CT considered the physical properties of amino acids and their dipole and side-chain volumes respectively. Then LD uses triples to describe composition, transition and distribution of sequence, while PseAAC further studies order information of sequences. We propose to combine different descriptors to achieve PPI prediction to obtain more information from protein interactions.

Ensemble learning is a machine learning method, which uses a series of learners and uses some rules to integrate the learning results so as to obtain better performance than a single learner. And ensemble learning has broad application prospects in many fields such as protein phosphorylation site prediction, genome function prediction and cancer prediction in bioinformatics (Gomes et al., 2017; Krawczyk et al., 2017). The previous works also demonstrate the effectiveness of classifier ensemble and provide some guidelines to generate an ensemble classification model (Martin et al., 2005; Han and Huang, 2006; Huang and Zheng, 2006; Huang and Du, 2008). Wang used a boosting technique to generate multiple classifiers iteratively to solve the problem of imbalance between positive and negative data when predicting the phosphorylation sites (Wang et al., 2017). Wang took a random forest and voting method as a basic classifier integration strategy separately to predict PPI sites (Wang et al., 2019). You et al. (2019) chose the basic classifiers with optimal performance, leaving the classifiers with small differences and using the max-wins voting (MWV) strategy to predict DNA binding proteins. Zhang et al. (2019a) trained 27 models by combining AC, MCD, LD with 9 DNN models of different configurations, and integrated these models through Double-layers BP Neural Network.

Furthermore, when exploring protein interactions and interaction networks, it is nonnegligible to quantify the interaction/non-interaction relationship between two proteins. One solution is to directly concatenate the features of the two proteins to form a feature vector (Zhang et al., 2019b), which lacks the information characteristics of the interaction/non-interaction between two proteins; another solution is to extract two features with two different networks and combine the features to form a new feature vector as the input of the model (Du et al., 2017b; Hashemifar et al., 2018), which is incapable of learning inherent relation of the proteins. Recently in natural language processing domain, researches have shown that attention mechanisms can effectively emphasize the relatively important parts of the input sentences and help boost the performance of relation extraction (Chen et al., 2017; Du et al., 2017a). In bioinformatics, attention mechanism is also adopted in chemical-protein interaction (CPI) (Zhang et al., 2019b), kinase-specific phosphorylation site prediction (Wang et al., 2017) and so on. In Xuan et al. (2019) model, exploiting the attention mechanism module to learn features or extract the relationship between IncRNA and disease provides more information. Wang et al. (2017) designed a two-dimensional independent attention mechanism for predicting phosphorylation sites, which enabled the model, called MusiteDeep, to automatically search important positions of the output sequences to estimate the contribution of each element in the sequences and feature dimensions. However, the above researches concern only single attention mechanism in the deep neural network model, which can be replaced by the multi-head attention mechanism that can exert attention multiple times and divide attention information into multiple heads. Liu et al. (2018) integrated attention pooling into the gated recurrent unit (GRU) model to extract CPIs. Verga et al. (2018) combined the Multi-head attention with convolution neural networks to construct a transformer model to extract the document-level biomedical relations. Thus, a multi-head attention mechanism will make it easier to capture the relevant important information for deep neural networks in PPI extraction.

Motivated by attention mechanisms and ensemble learning, we propose an algorithm called EnAmDNN, which at first extracted the biophysical-chemical information of protein sequences through AC, CT, LD, and PseAAC and association with the interactive description of each protein in protein interaction network; then it automatically extracted the protein features by multi-layer convolutional neural network, adopted attention mechanism to analyze deep-seated relationship of proteins and then forms the feature vectors. In EnAmDNN, 16 kinds of DNN models are trained through 4 characteristic bases which are the inputs of 4 DNNs with different layers and different neurons. In the integration module, the outputs of 16 DNNs are taken as the inputs of deep neural networks finally, and the five-fold cross validation is adopted to comprehensively predict protein interactions and interaction networks. Our contributions can be summarized as follows: (1) the new network structure can automatically extract highly abstract representations and detect the sequence specificity of proteins; (2) the attention mechanism is adopted to analyze internal links between the two proteins and the network description of each protein, instead of directly concatenating the two proteins, to improve the prediction accuracy;(3) ensemble learning considers the advantages of different descriptors and different DNNs to achieve comprehensive learning.

## Preliminaries

### Deep Neural Network

It turns out that deep neural network (DNN) plays an important role in bioinformatics (Alipanahi et al., 2015; Zhou and Troyanskaya, 2015; Liu et al., 2016), i.e., predicting inner-organization and trans-organization RNA splicing patterns (Leung et al., 2014). DeepMind applied DNN to the detection of sequence specificity of the DNA-RNA binding protein, which is superior to other methods (Alipanahi et al., 2015); DeepSEA applied DNN to learn the code of regulatory sequences from chromatin map sequences in order to discern priorities of other functional varieties (Zhou and Troyanskaya, 2015); other examples include genome informatics extraction, detection of protein structure and medicine discovery. In short, compared with other sequence-based methods, DNN has the following advantages: (1) it can automatically learn certain protein sequences; (2) it can reduce the influence of noise on the raw data and extract the hidden high dimension representation (Bengio et al., 2013). However, the performance of DNN is closely related to the network configuration and may vary greatly for different configurations.

### Protein Representation Technique

Different representation techniques of protein features may have a strong impact on the performance of PPI prediction, making it a challenge to effectively express the protein features and describe the connections of two proteins. We choose four representative protein techniques instead of one to avoid the limitation brought by a single technique.

#### Auto Covariance Technique

Two proteins interact with each other through electrostatic, hydrophobic, steric and hydrogen bond, which can be reflected by the seven physicochemical properties of amino acids, including hydrophobicity (H_1_), hydrophilicity (H_2_), volumes of side chains of amino acids (VSC), polarity (P_1_), polarizability (P_2_), solvent-accessible surface area and net charge index of side chains. The above properties are exploited by the auto-covariance method to transform amino acid sequence into uniform matrices which reveal the special connection of two residues under a certain distance and are widely applied in protein- encoding. For example, a protein sequence of length *L* is calculated as follows (Guo et al., 2008):

(1) $$\begin{array}{l} AC(lag,j)=\frac{1}{L-lag}\sum_{i=1}^{L-lag}\left(X_{i,j}-\frac{1}{L}\sum_{i=1}^{L}X_{i,j}\right)\times (X_{(i+lag),j}\\ \;-\frac{1}{L}\sum_{i=1}^{L}X_{i.j}) \end{array}$$

*Xij* represents the *j*-th physical property of the *i*-th amino acid in the protein sequence; *lag* represents the distance between residues; then proteins of various lengths are encoded as vectors of equal length *lg* ^*^ p, where lg is the maximum *lag* (lag = 1, 2, …, lg), p is the number of physical properties. In this study, p was 7, reflecting the characteristics of the seven amino acids. As with Guo, we set the log to 30 (Guo et al., 2008). Therefore, each protein sequence is represented as a 210-dimensional vector.

#### Conjoint Triad Technique

**Figure 1:**
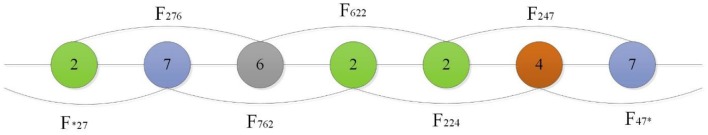
For a grouped sequence “2762247,” the numerical code string of consecutive amino acids are “276,” “762,” “622,” “224,” “247,” and “^*^27,” “47^*^” according to Shen, and “^*^” is considered to be the first or second amino acid of an amino acid in a continuous amino acid. So its triad types are F276, F762, F622, F224, F427.

Shen et al. (2007) introduced a conjoint triad technique to represent sequence information of each protein, in which any three contiguous amino acids are regarded as a unit and the characteristics of one amino acid and its vicinal amino acids are fully considered. First, the conjoint triad divides 20 standard amino acids into 7 groups according to their dipole and side-chain volumes, then the triads can be distinguished according to the type of amino acid. According to Shen's settings, there are 343 (7 × 7 × 7) triad types (Shen et al., 2007), as shown in Figure 1.

Finally, the PPI information of protein sequences are projected into the homogeneous vector space according to the frequency of each triad type, where each protein is represented by a 343-dimensional vector.

#### Local Descriptor Technique

**Figure 2:**
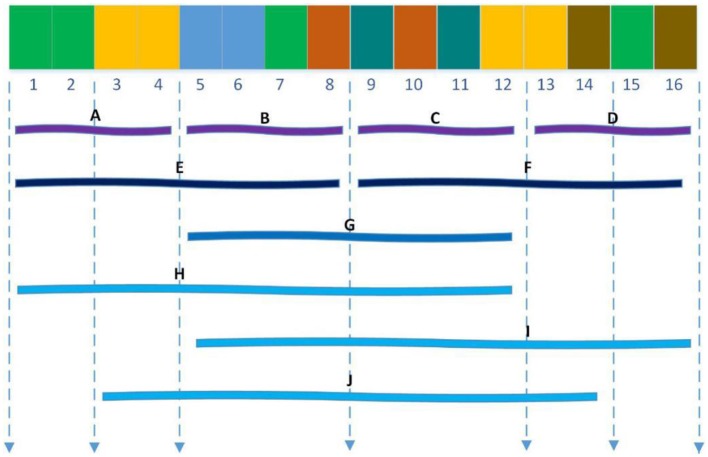
Ten regions **(A–J)** of the entire protein sequence. The regions **(A–D)** are generated by dividing the whole sequence into four equal regions, and regions **(E,F)** are generated by dividing the whole sequence into two equal regions. The region **(G)** stand for the central 50% of the entire sequence. And the regions **(H–J)** stand for the first 75%, the final 75% and the central 75% of the entire sequence respectively.

The Local descriptor technique (Zhou et al., 2011) also divided 20 standard amino acids into 7 groups as shown in Table 1 and divided the entire protein sequence into 10 regions as shown in Figure 2. For each sub-sequence, three descriptors, composition (C), transition (T) and distribution (D), are applied to describe its trait where C represents the proportion of each amino acid group; T represents the frequency with which amino acids in one group are followed by amino acids of another group; D measures the proportion of chain length where the top 25, 50, 75, and 100% of the amino acids of a particular group are located. For the local descriptor method, each region produces 63 values, where C represents 7, T represents 7, and D represents 35, and then each protein is encoded as a 630-dimensional vector.

**Table 1:** Division of amino acid into seven groups based on the dipoles and volumes of the side chains.

**Group 1**	**Group 2**	**Group 3**	**Group 4**	**Group 5**	**Group 6**	**Group 7**
A, G, V	C	F, I, L, P	M, S, T, Y	H, N, Q, W	K, R	D, E

For example, according to Table 1, the sequence “ACLACLCCLAALLCCCLALALAAALL” is converted into the amino acid group “1231232231133232131131311133133” so that the sub-sequence contains 9 “1”, 7 “2,” and 10 “3.”For feature C, 9/(9 + 7 + 10) = 0.3461, 7/(9 + 7 + 10) = 0.2693, 10/(9 + 7 + 10) = 0.3846; for feature T, there are 2 cases that “1” is converted to “2” or “2” is converted to “1,” then 2/25 = 0.08; similarly, transitions between “3” and “1” as well as “2” and “3” are 3/25 = 0.12, 6/25 = 0.24, respectively; for feature D, there are nine “1”s, then the D descriptor for 1 is 1/26 = 0.0384, [0.25^*^9 + 0.5]/26 = 0.0769, [0.5^*^9 + 0.5]/26=0.1923, [0.75^*^9 + 0.5]/26 = 0.2692, [1^*^9 + 0.5]/26 = 0.3462.

#### Pseudo Amino Acid Composition (PseAAC) Technique

Tian et al. (2019) used a sequence encoding technique based on pseudo amino, that is, PseAAC. Given a protein sequence P with *L* amino acid residues:

$$\begin{array}{l} S_{1}S_{2}S_{3}S_{4}\ldots .\ldots S_{L} \end{array}$$

where *S*_*i*_ represents the *i*th residue of the protein P, 1 ≤ *i* ≤ *L*.

According to the PseAAC technique, the protein P can be formulated as

(2) $$\begin{array}{l} P={\left[x_{1},x_{2},\ldots ,x_{20},x_{21},\ldots ,x_{20+\lambda }\right]}^{T},\;\left(\lambda <L\right) \end{array}$$

where the 20 + λ components are given by

(3) $$x_{k}=\{\begin{array}{c} \frac{f_{k}}{\sum_{i=1}^{20}f_{i}+\omega \sum_{j=1}^{\lambda }\theta_{j}},(1\leq k\leq 20)\\ \frac{\omega \theta_{k-20}}{\sum_{i=1}^{20}f_{i}+\omega \sum_{j=1}^{\lambda }\theta_{j}},(21\leq k\leq 20+\lambda ) \end{array}$$

In Equation (3), *f*_*k*_(*k* = 1, 2, …, 20) are the normalized occurrence frequencies of 20 amino acids in protein P; ω is the weighting factor set to 0.05 in general work; and θ_*j*_(*j* = 1, 2, …, λ) denotes the order relationship between two residues that are *j* residues apart, which is shown as follows:

(4) $$\begin{array}{l} \theta_{j}=\frac{1}{L-j}\overset{L-j}{\underset{i=1}{\sum }}J_{i,i+j}\left(j<L\right) \end{array}$$

(5) $$\begin{array}{l} J_{i,i+j}=\frac{1}{3}\overset{3}{\underset{p=1}{\sum }}\left[H_{p}\left(A_{i+j}\right)-H_{p}\left(A_{i}\right)\right] \end{array}$$

where *J*_*ij*_denotes the order relationship function between amino acid *A*_*i*_ and *A*_*j*_, *H*_*p*_(*A*_*i*_) denotes the *p*th property of *A*_*i*_. *H*_1_(*A*_*i*_),*H*_2_(*A*_*i*_) and *H*_3_(*A*_*i*_) are the hydrophobicity value, hydrophilicity value and side-chain mass for the amino acid, respectively. This coding method contains more sequence characteristics because it considers not only the physicochemical properties of the protein but also the order information of sequences.

## Materials and Methods

### Data Sets

We collect the dataset information of Parkinson's disease (PD), Alzheimer's disease (AD), cancer, cardiac and diabetes, whose interactive information is from IntAct database (Kerrien et al., 2007) and sequence information from Uniprot (Bairoch et al., 2004). We are concerned about positive-negative selection in our work. For the positive set, proteins and protein pairs that contain less than 50 amino acids and 40% of sequence identity are removed to eliminate the variance caused by minor bias proteins to the model. The negative set was obtained by pairing proteins whose subcellular localization is different (Guo et al., 2008) or GO Cellular Component (CC) and Biological Process (BP) ontology with experimental evidence codes (Muley and Ranjan, 2012). The subcellular location information on the proteins is extracted from Uniprot. According to this information, a protein can be divided into several types of localization cytoplasm, nucleus, mitochondrion, endoplasmic reticulum, Golgi apparatus, peroxisome and vacuole. The way to construct negative set must meet the following requirements: (1) the non-interacting pairs cannot appear in the positive data set; (2) the contribution of proteins in the negative set should be as harmonious as possible. In our work, the ratio between positive and negative set is 1:1, where the negative sets are randomly chosen from non-interactive pairs.

Finally, we have five independent PPI datasets: Parkinson's disease (PD) (4,127 interacting pairs and 4,127 non-interacting pairs), Alzheimer disease (AD) (4,096 interacting pairs and 4,096 non-interacting pairs), Cancer (6,352 interacting pairs and 6,352 non-interacting pairs), Cardiac (2,663 interacting pairs and 2,663 non-interacting pairs) and Diabetes (163 interacting pairs and 163 non-interacting pairs).

### Evaluation Criteria

The following metrics are taken into account to perform evaluation: Overall Prediction Accuracy, Recall, Precision, F_1_ score values, and Area under the ROC Curve (AUC) (Zhang et al., 2019a). The first four metrics are defined as follows:

(6) $$\begin{array}{l} \text{Accuracy}=\frac{\text{TP}+\text{TN}}{\text{TP}+\text{TN}+\text{FP}+\text{FN}} \end{array}$$

(7) $$\begin{array}{l} \text{Recall}=\frac{\text{TP}}{\text{TP}+\text{FN}} \end{array}$$

(8) $$\begin{array}{l} \text{Precision}=\frac{\text{TP}}{\text{TP}+\text{FP}} \end{array}$$

(9) $$\begin{array}{l} {\text{F}}_{\text{1}}=\frac{\text{2TP}}{\text{2TP}+\text{FP}+\text{FN}} \end{array}$$

where TP (true positive) is the number of true interacting pairs found in the positive data set, TN (true negative) is the number of true non-interacting pairs with correct prediction, FP (false positive) is the number of the predicted interacting pairs not found in the positive data set, and FN (false negative) is the number of the true interacting pairs with false prediction.

### Ensemble Deep Neural Networks

**Figure 3:**
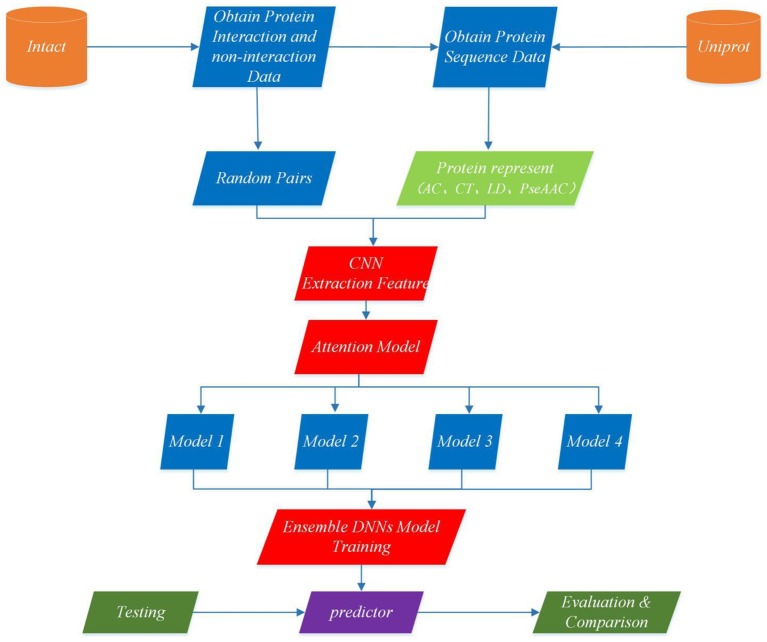
Flowchart of EnAmDNN for predicting protein-protein interactions. First, the interaction pairs and non-interaction pairs of related proteins are obtained from IntAct, and all protein sequence data of UniProt are obtained; the appropriate proportion of interaction pairs and non-interaction pairs are selected, and each group of protein pairs (including interaction pairs and non-interaction pairs) is vectorization by AC, CT, LD, and PseAAC techniques; put vector protein into convolution neural network for feature extraction of each protein; extracted features are transferred to attention mechanism module for deep analysis of interaction between each group of protein pairs; then the analyzed features are input into deep neural network of different models for training; finally, the final prediction results are obtained by integrating the prediction results of different models.

This section describes EnAmDNN model that predicts PPI based on protein sequences, which consists of the input module, the convolution module, the attention mechanism module, the DNN module and the integration module. Each protein sequence is encoded, by the input module, through the protein representation techniques, as a vector, whose feature is extracted by the convolution module. Then, the internal link in the protein pair is detected through the attention module, and then the analyzed protein pair is provided to 16 dependent learners. After the training is completed, these learners will be integrated through a two-layer hidden layer neural network. The working process of an EnAmDNN is shown as Figure 3.

#### Deep Convolutional Module

The convolution module is a batch of normalized layers, a stack of convolutional layers and activation layers, which can automatically extract features of vectorized protein sequences. In our model, the output of the convolution module is calculated by an expression that starts with a convolution layer and ends with a convolution layer:

(10) $$\begin{array}{l} In_{t+1}=RELU\left(Batch_{\beta ,\delta }\left(Conv_{\lambda }\left(In_{t}\right)\right)\right) \end{array}$$

(11) $$\begin{array}{l} Out=Conv_{\gamma }\left(In_{n}\right) \end{array}$$

Repeat *In* n times and enter the result *In*_*n*_ into Equation 11. Where *Out* is the output vector, *In* is the input vector and β, γ, δ, λ are the parameters of batch normalization and convolution layers.

The convolution layer searches the sequences according to their input orders and outputs the corresponding features; the batch normalization layer takes in the feature vectors and normalizes their mean values and the variances; ReLU layer takes in the normalized vectors and introduces non-linearity to achieve complex function approximation. Then the above processes repeat n times to obtain the feature vector.

#### Attention Module

**Figure 4:**
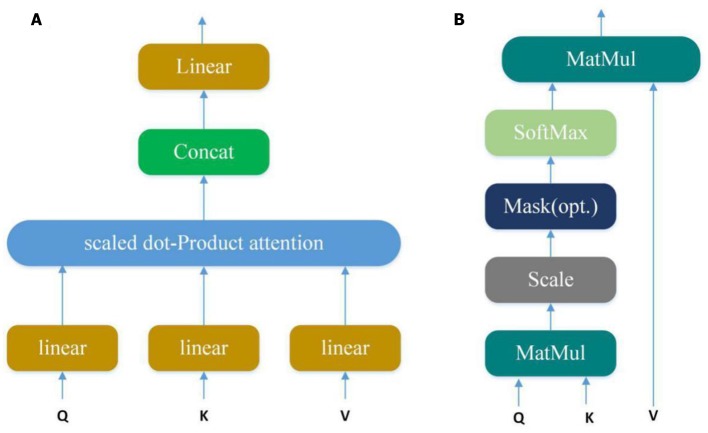
**(A)** Multi-Head Attention consists of several attention layers. (Vaswani et al., 2017) First, query, key and value go through a linear transformation, and then enter them into scaled dot-Product attention to generate many heads; then concatenate these heads to keep relevant information in different representation subspaces. **(B)** Scaled dot-product attention (Vaswani et al., 2017). Obtain weights by similarity calculation between query and each key, and the weights are normalized by softmax function; then attention is obtained by the weight and the corresponding value.

Convolution layer can automatically learn potential features from input sequences, but only a small part of these potential features are very important in PPI. In our model, we use the multi-attention mechanism to adjust the weight of the input sequences to further emphasize the relatively crucial features. Applying the attention multiple times may learn more important features than single attention and allowing the model to learn relevant information in different representation subspaces (Vaswani et al., 2017). It can be understood that attention selectively selects a small amount of important information that is beneficial to PPI from a large amount of information and focuses on important information, ignoring most of the insignificant information. We choose the mechanism of multiple attention rather than directly connecting the two protein eigenvectors to increase the exploration of protein pairs and further use the indirect relationship between residues to obtain more accurate information. The Multi-head attention calculates the output based on the query and a set of key-value pairs, where *Q, K, V* denote query, key, and value respectively. The specific structure is shown in Figure 4:

Query, key and value go through a linear transformation first, and then enter them into scaled dot-Product attention. At this time, the attention calculation formula is as follows:

(12) $$\begin{array}{l} \text{Attention}\left(Q,K,V\right)=\text{softmax}\left(\frac{QK^{\prime }}{\sqrt{d_{k}}}\right)V \end{array}$$

Where $\sqrt{d}$ is scaling factor. The core of the Multi-head attention is employing the above attention multiple times, and one time attention means one head. Suppose the Multi-head attention needs to be done *h* times to generate *h* heads, the Att_head_ can be calculated as follows:

(13) $$\begin{array}{l} Att_{headi}\left(Q,K,V\right)=Attention\left(QW_{i}^{Q},KW_{i}^{K}VW_{i}^{V}\right),1<i\leq h \end{array}$$

where $W_{i}^{Q},W_{i}^{K},W_{i}^{V}$ are parameter matrices. Finally, these heads are concatenated and once again linearly transformed by

(14) $$\begin{array}{l} MultiHead\left(Q,K,V\right)=Linear\left(Concat\left(Att_{head\text{1}},\ldots ,Att_{head\text{h}}\right)W^{\mu }\right) \end{array}$$

In order to keep the invariance of features, we introduce average pooling and maximum pooling to reduce the errors caused by model parameters and retain information of global and local features.

(15) $$\begin{array}{l} newMultiHead(Q,K,V)\\ =Concat(AvgPool(MatMul(MultiHead(Q,K,V),Q)),\\ MaxPool(MatMul(MultiHead(Q,K,V),Q))) \end{array}$$

where *AvgPool* is the function of average pooling and *MaxPool* is the function of maximum pooling.

For a protein pair (P_1_, P_2_), it is expressed as *S*_1_, *S*_2_ respectively after convolution layer. We use the merge layer to fuse the protein pairs that are redistributed by the attention mechanism. The calculation formula of the merge layer is as follows:

(16) $$\begin{array}{l} {S_{1}}^{\prime }=newMultiHead\left(S_{1},S_{2},S_{1}\right),\\ {S_{2}}^{\prime }=newMultiHead\left(S_{2},S_{1},S_{2}\right) \end{array}$$

(17) $$\begin{array}{l} Merge\left({S_{1}}^{\prime },{S_{2}}^{\prime }\right)=Concat\left(\frac{{S_{1}}^{\prime }\cdot {S_{2}}^{\prime }}{\left|{S_{1}}^{\prime }|\times |{S_{2}}^{\prime }\right|},{S_{1}}^{\prime }A{S_{2}}^{\prime },{S_{1}}^{\prime },{S_{2}}^{\prime }\right) \end{array}$$

where *A* is weight.

The basic model of our work is mainly constructed by 3.3.1 the deep convolution module, 3.3.2 the attention mechanism module and the deep neural network. The specific basic learning algorithm is shown in algorithm 1.

**Algorithm 1:** Basic learning algorithm based on multi-head attention with pooling

**Input:** interaction data $D=\;\left\{\;x_{i},y_{i}\;\right\}{\;}_{i=\text{1}}^{n}$, encoded protein sequence pair*x*_*i*_ = (*s*_*i*1_, *s*_*i*2_)
**Return:** basic learning algorithm parameters W, b
1: **for** *i* = 1 to *n*: 2: *S*_1_, *S*_2_ ← *x*_*i*_(*s*_*i*1_, *s*_*i*2_) represent encoded protein p_1_ and protein p_2_ 3: **for** *iter* = 1 to *t*: 4: *S*_1, *iter*+1_ = *RELU* (*Batch*_β, δ_(*Conv*_λ_(*S*_1, *iter*_))) 5: *S*_2, *iter*+1_ = *RELU* (*Batch*_β, δ_(*Conv*_λ_(*S*_2, *iter*_))) 6: **end for** 7: *S*_1_ ← *Conv*_γ_(*S*_1, *t*_) 8: *S*_2_ ← *Conv*_γ_(*S*_2, *t*_) 9: calculate the importance value of single attention by $$\begin{array}{l} Attenhead_{S_{\text{1}}}^{i}=\text{softmax}\left(\frac{S_{1}{S_{2}}^{\prime }}{\sqrt{d_{k}}}\right)S_{1},\\ Attenhead_{S_{\text{2}}}^{i}=\text{softmax}\left(\frac{S_{\text{2}}{S_{\text{1}}}^{\prime }}{\sqrt{d_{k}}}\right)S_{\text{2}} \end{array}$$ 10: connect multiple attentions by $S_{{}_{1}}^{\text{*}}=MultiHead\left(S_{1},S_{2},S_{1}\right)$,$S_{{}_{\text{2}}}^{\text{*}}=MultiHead\left(S_{\text{2}},S_{\text{1}},S_{\text{2}}\right)$ 11: Calculate new feature importance value of multi-head attention with pooling by $$\begin{array}{l} S_{\text{1}}^{\text{**}}=newMultiHead\left(S_{1}^{*},S_{2}^{*},S_{1}^{*}\right),\\ S_{\text{2}}^{\text{**}}=newMultiHead\left(S_{\text{2}}^{*},S_{\text{1}}^{*},S_{\text{2}}^{*}\right) \end{array}$$ 12: merge the sequence feature pairs as the input of the network by $Concat\left(\frac{S_{\text{1}}^{\text{**}}\cdot S_{\text{2}}^{\text{**}}}{\left|S_{\text{1}}^{\text{**}}|\times |S_{\text{2}}^{\text{**}}\right|},S_{\text{1}}^{\text{**}}AS_{\text{2}}^{\text{**}},S_{\text{1}}^{\text{**}},S_{\text{2}}^{\text{**}}\right)$ 13: *output* = softmax(*W* · *merge* + *b*) 14: **end for** 15: **Return** *W, b*

#### Ensemble Strategy

Ensembles of independent deep neural networks can improve the performance of a single network (Bairoch et al., 2004). In the Otto group product classification challenge, the first one won the championship by stacking 30 models. The model achieved remarkable results, and we also adopted the stacking method of ensemble learning in our work. Secondly, to predict the effect better, the trainers of each primary model keep stability and diversity as much as possible.

**Figure 5:**
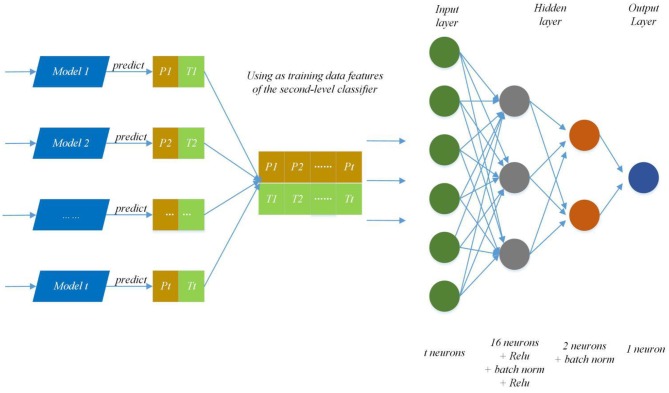
Ensemble strategy composed of deep neural networks. The first layer results (P_n_, T_n_) (0 < n < t+1) are predicted by T primary learners, where P_n_ and T_n_ stand for training data and the prediction result; then use recombined (P_n_, T_n_) as training data features of the second-level classifier and put it into deep neural networks to predict protein interaction networks.

We modify the internal structure of the algorithm and learn from different feature representations, which are two strategies to maintain diversity and achieved improvement, so we also take the same measures (Zhang et al., 2019a). In practice, we choose four feature representations to quantify the characteristics of each protein and set different parameters of DNNS according to the characteristics of each representation. Then we use the stacking method to combines with five-fold cross validation, the primary learners are trained from the initial data set, and a new data set is generated by the primary learners for training the secondary learner. It means the output of each primary trainer is input as an example to the secondary trainer for fusion output and PPI prediction. Here, the secondary trainer is composed of deep neural networks. Its structure is shown in Figure 5 and ensemble strategy is described in algorithm 2.

## Result

### Comparing the Prediction Performance With Other Methods

All the experiment were carried out on a computer with CentOs, Cuda version 10.1.243, CuDnn version 7.0 and software environment python3.7+keras2.0+torch1.3.

**Algorithm 2:** EAM algorithm

**Input:** interaction data; basic learning algorithm ψ_1_,ψ_2_,…,ψ_M_; L layers; learning rate η; Max iterations and iteration threshold ε; class k ∈ {0, 1}
**Return:** ensemble strategy parameters *W, b*
1: initialize weight matrix *W* and Bias *b, iter as* 1 2: extract protein sequence from uniprot 3: vectorize protein sequence by AC, LD, CT, PseAAC 4: divide training data $TrD={\left\{D_{i}\right\}}_{i=1}^{K}={\left\{x_{i},y_{i}\right\}}_{i=1}^{n}$ and testing data *TeD* 5: **for** *m* = 1 to *M* do 6: **for** *k* = 1 to *K* do 7: train $h_{m}=\phi_{m}\left({\bar{D}}_{k}\right)$ with ${\bar{D}}_{k}$ 8: predict *D*_*k*_ and require predicted value *P*_*mk*_ = *h*_*m*_(*D*_*k*_) 9: predict *T* and and require predicted value *T*_*k*_ = *h*_*m*_(*T*) 10: **end for** 11: splicing predicted value of training data through *m*th basic model by $P_{m}={\left(P_{m1},P_{m2},\ldots ,P_{mK}\right)}^{T}$ 12: splicing predicted value of testing data through *m*th basic model by *T*_*m*_ = (*T*_1_ + *T*_2_ + … + *T*_*K*_)/*K* 13: **end for** 14: construct new training data by 15: and construct new testing data by *newT* = (*T*_1_, *T*_2_, …, *T*_*M*_) 16: **Repeat:** 17: *iter* + 1 18: **for** i = 1 to *n*: 19: set $a^{\left(1\right)}=x_{i}^{*}$ 20: for *l*=2 to *L*: *a*^*l*^ = σ(*W*^*l*^*a*^*l*−1^ + *b*^*l*^) 21: calculate probability *p*_*k*_ divided into class k 22: calculate $loss^{L}=-\sum_{k=0}^{1}y_{k}^{L}\cdot \log(p_{k})$ 22: for *l*=2 to *L*: 23: **end for** 24: for *l*=2 to *L*: 25: $\nabla W^{l}=\mu \sum_{i=1}^{n}loss^{l}{\left(a^{l-1}\right)}^{T},\nabla b^{l}=\mu \sum_{i=1}^{n}loss^{l}$ 26: *W*^*l*^ ← *W*^*l*^ − ∇*W*^*l*^, *b*^*l*^ ← *b*^*l*^ − ∇*b*^*l*^ 27: **end for** 27: Until ∇*W*, ∇*b* < ε 28: **Return** *W, b*

In order to evaluate the performance of EnAmDNN, we compared it with the approaches proposed by Guo et al. (2008), Zhou et al. (2011), Du et al. (2017b), Yang et al. (2010), Zhang et al. (2019a) emphasized the highest score of each evaluation criteria in bold and present the results in Tables 2–6, which separately utilize AC, LD, CT, APAAC, PseAAC to encode amino acid sequence, and predicted PPI with SVM, k-nearest neighbor (kNN) or DNNs, all of which share the same training sets and the same testing sets. It can be seen from Table 2 that EnAmDNN generally outperforms these predictors, where EnAmDNN achieved optimal prediction performance in all the datasets, especially in AD, with an accuracy of 94.67%, and a recall rate of 93.29%. The accuracy is 95.41%, F1 is 94.33%, and AUC is 94.63%. This is because, in EnAmDNN, feature representations in protein sequences are coordinated, and new features are obtained through different classifiers. Compared with the recent EnsDNN model, in five independent data sets, the AUC index DnAmDNN has increased by 0.89, 0.41, 0.61, 0.6, 3.90%, and the accuracy of PPI prediction are relatively high. The EnAmDNN model takes advantage of the multi- head attention mechanism, that is, extracts the internal links of the PPI, thereby improving the performance of the model.

**Table 2:** AD with various methods.

**Methods**	**Accuracy**	**Recall**	**Precision**	**F1**	**AUC**
EnsAmDNN	**0.9467**	**0.9329**	**0.9541**	**0.9433**	**0.9463**
SVM_AC	0.7886	0.6369	0.8956	0.7435	0.7831
kNN_LD	0.7549	0.7507	0.7602	0.7542	0.7556
SVM_LD	0.804	0.6735	0.9174	0.7754	0.805
NDDs_APAAC	0.898	0.9093	0.8907	0.8993	0.8977
EnsDNN	0.9372	0.9255	0.9531	0.9388	0.938

*The highest score of each evaluation criteria is emphasized in bold*.

**Table 3:** PD with various methods.

**Methods**	**Accuracy**	**Recall**	**Precision**	**F1**	**AUC**
EnsAmDNN	**0.895**	**0.8568**	0.9275	**0.8903**	**0.8951**
SVM_AC	0.797	0.62	0.9373	0.7444	0.7904
kNN_LD	0.8057	0.8042	0.7906	0.7958	0.8064
SVM_LD	0.8315	0.7337	0.9084	0.8108	0.831
NDDs_APAAC	0.8773	0.8247	0.9058	0.8627	0.8906
EnsDNN	0.8917	0.8433	**0.9311**	0.8846	0.8915

*The highest score of each evaluation criteria is emphasized in bold*.

**Table 4:** Cancer with various methods.

**Methods**	**Accuracy**	**Recall**	**Precision**	**F1**	**AUC**
EnsAmDNN	**0.8502**	**0.8062**	**0.8863**	**0.8436**	**0.8508**
SVM_AC	0.6524	0.4848	0.7347	0.5811	0.6545
kNN_LD	0.6475	0.6761	0.6202	0.6458	0.6471
SVM_LD	0.6673	0.5591	0.715	0.6263	0.6681
NDDs_APAAC	0.7551	0.7224	0.7764	0.7474	0.7555
EnsDNN	0.8008	0.7549	0.8362	0.7925	0.802

*The highest score of each evaluation criteria is emphasized in bold*.

**Table 5:** Cancer with various methods.

**Methods**	**Accuracy**	**Recall**	**Precision**	**F1**	**AUC**
EnsAmDNN	**0.907**	0.8523	**0.96**	**0.9018**	**0.9088**
SVM_AC	0.7356	0.6316	0.806	0.7037	0.7371
kNN_LD	0.7671	0.7246	0.7934	0.7565	0.7675
SVM_LD	0.7819	0.7545	0.7962	0.774	0.7816
NDDs_APAAC	0.8454	0.8326	0.837	0.8339	0.8446
EnsDNN	0.9039	**0.8747**	0.9252	0.899	0.9034

*The highest score of each evaluation criteria is emphasized in bold*.

**Table 6:** Diabetes with various methods.

**Methods**	**Accuracy**	**Recall**	**Precision**	**F1**	**AUC**
EnsAmDNN	**0.8333**	0.871	0.7941	**0.8308**	**0.8355**
SVM_AC	0.7891	0.6667	0.7941	0.7128	0.7819
kNN_LD	0.7568	0.8571	0.75	0.8	0.7411
SVM_LD	0.7813	0.8269	0.7361	0.7742	0.7885
NDDs_APAAC	0.803	**0.9118**	0.7561	0.8267	0.7996
EnsDNN	0.8030	0.7576	**0.8333**	0.7937	0.8030

*The highest score of each evaluation criteria is emphasized in bold*.

**Figure 6:**
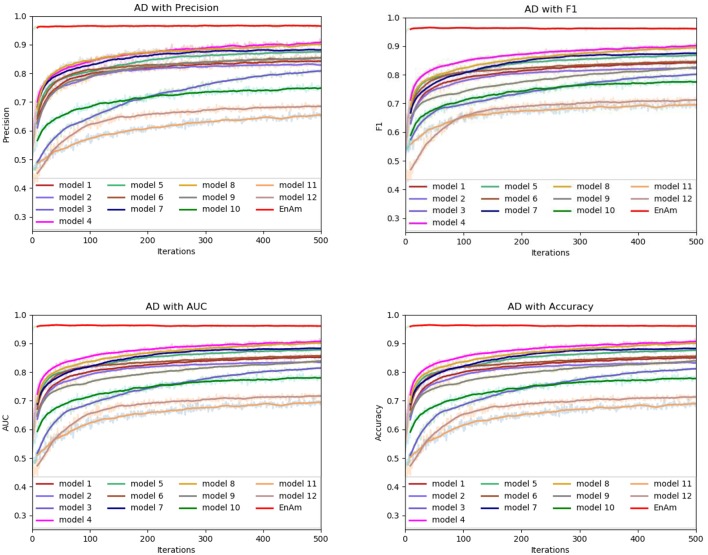
Comparison of evaluation indexes of each basic model and ensemble model with AD data set.

**Figure 7:**
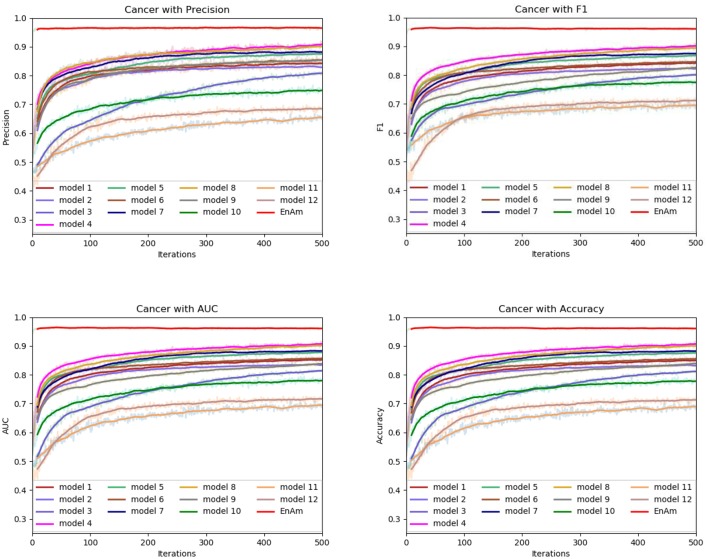
Comparison of evaluation indexes of each basic model and ensemble model with Cancer data set.

**Figure 8:**
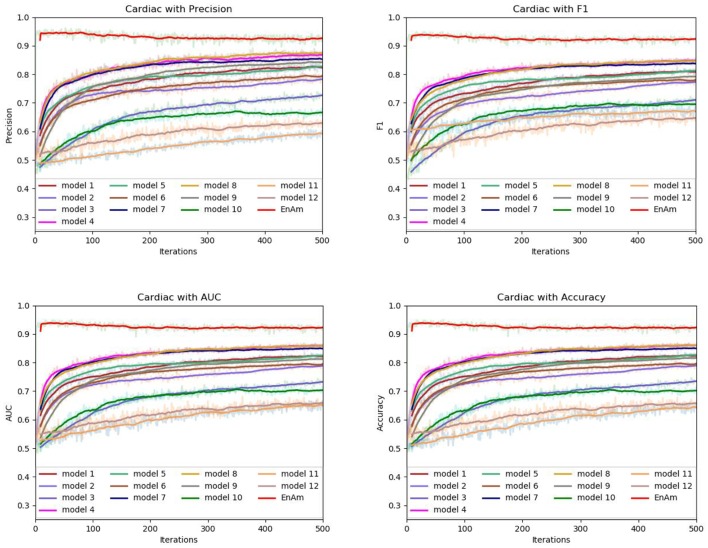
Comparison of evaluation indexes of each basic model and ensemble model with Cardiac data set.

**Figure 9:**
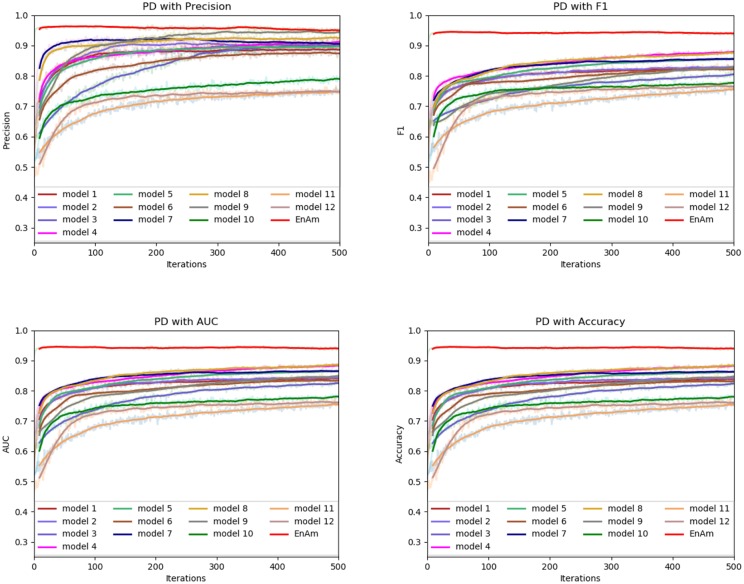
Comparison of evaluation indexes of each basic model and ensemble model with PD data set.

To further demonstrate the effect of ensemble strategy, five-fold cross-validation is employed to improve the reliability of the results. Figure 6 shows the performance of each basic learner, where it can be observed, taking AD dataset as an example, that each basic learner, associated with five-fold cross-validation method, shows fairish prediction performance, which is reflected on all the evaluation criteria Accuracy, Recall, Precision, F1, AUC. The result indicates that our model extracts and trains the features produced by basic learners and that the shortcoming of each basic learner is overcome to a certain degree. It's also confirmed with PD, Cardiac and Cancer in Figures 7–9.

### Performance of PPI Prediction

To further study the effectiveness of the ensemble strategy, we designed two different network architectures: (a) concatenating four feature representations (AC, LD, CT, PseAAC) as the input to the first layer classifiers (namely EnAm-Con) and (b) separately taking one feature representation as the input to the first layer classifiers (namely EnAm-Sep including EnsAC, EnsLD, EnsCT, EnsPseAAC). EnAm-Con first concatenates four feature representations and then integrated 12 trained DNNs in the same way as EnAmDNN. For EnAm-Sep, we separately trained 12-model DNNs based AC, LD, CT, and PseAAC, and integrated these DNNs in the same way as EnAmDNN. The performance of EnAm-Con and EnAm-Sep which emphasized in bold are also listed in Table 7 where it can be observed that the LD method performs better than AC and PseAAC method. The LD method of AUC value obtained from the first four data sets are 6.2, 4.89, 3.57, 7.17, 0.8 and are 16.37, 9.05, 14.19, 17.89, 9.46% higher than AC and PseAAC methods separately, which is because LD can encode more interaction information. It can be seen from Table 6 that EnAm-Con performs better than EnAm-Sep, proving that concatenating different feature representations as new feature vector can improve the accuracy of the ensemble strategy. It can be concluded that these four representations are complementary to each other and our ensemble strategy is effective and feasible.

**Table 7:** Comparison of EnAm-Con and EnAm-Sep.

**Data sets**	**EnsAC**	**EnsLD**	**EnsCT**	**EnsPseAAC**	**EnsCon**	**EnAMDNN**
AD	0.8502	0.9029	0.8949	0.7759	0.9338	**0.9456**
PD	0.8378	0.8788	0.8820	0.8059	**0.9242**	0.8951
Cancer	0.7638	0.7911	0.7889	0.6928	0.8468	**0.8508**
Cardiac	0.8201	0.8789	0.8888	0.7455	0.9020	**0.9088**
Diabetes	0.7516	0.7576	0.7917	0.6921	0.8459	**0.8787**

*The highest score of each evaluation criteria is emphasized in bold*.

**Figure 10:**
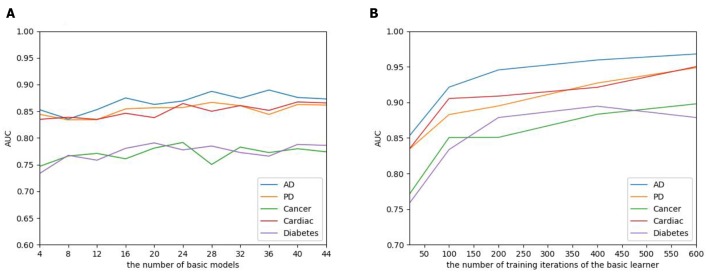
**(A)** The influence of the number of basic learners on the EnAmDNN. It can be observed that the AUC of the EnAmDNN tends to be stable when the number reaches 16. **(B)** The influence of the number of training iterations of the basic learners on the EnAmDNN. It can be seen that the model tends to remain stable at the point of 200.

The number of basic learners greatly influences the overall prediction performance, where the efficiency of the model continues to grow as the number of learners increases, to a point that the performance tends to be stable. To evaluate the influence of the EnAmDNN, we assign different numbers of DNNs to protein represent technique, such as 1, 3, 5, 7, 9. The result is presented in Figure 10, where it can be observed that the AUC of the EnAmDNN tends to be stable when the number reaches 16. The efficiency of the EnAmDNN may also be affected by the performance of each basic learner, for which we prepare 16 basic learners, iterate them for 600 times, and combine them through deep neural network. The result is shown as follows. It can be seen that the prediction performance improves as the iteration continues and the model tends to remain stable at the point of 200.

Table 8 reports the process running time of EnAmDNN based on fold cross-validation with 16 basic learners and iterate each basic learner for 600 times.

**Table 8:** Running time of EnAmDNN based on fold cross-validation.

**Date sets**	**AD**	**PD**	**Cancer**	**Cardiac**	**Diabetes**
Time (s)	1,273,589.80	835,292.29	2,618,297.11	1,145,029.32	101,784.60

Meanwhile, to further investigate the contribution of using an ensemble predictor with fold cross-validation, we integrated the simplified EnAmDNN, which don't use fold cross-validation. To reduce the impact of data dependency in the experiment, we constructed data sets on Cardiac based on Muley and Ranjan (2012) to observe the performance of proposed model. From Table 9, we can see that EnAmDNN achieves competent prediction performance with an average accuracy of 85.66%, precision of 89.47%, F_1_ of 85.16%, and AUC of 85.76. It has better performance than simplified EnAmDNN across evaluation metrics. The prediction results show that EnAmDNN with fold cross-validation is capable of predicting PPIs.

**Table 9:** Comparison of EnAmDNN and simplified EnAmDNN.

**Models**	**Accuracy**	**Recall**	**Precision**	**F1**	**AUC**
EnAmDNN	0.8749	0.8138	0.9337	0.8688	0.8765
Simplified EnAmDNN	0.8566	0.8142	0.8947	0.8516	0.8576

## Conclusions

In this paper, we propose an ensemble deep learning framework (EnAmDNN) with an attention mechanism that aims to predict protein interaction networks. EnAmDNN firstly extracts the feature information of protein sequences through AC, LD, CT, and PseAAC, and projects the information into various feature spaces to segment information of AC, LD, CT, PseAAC amino acid from different perspective; then the multi-head attention mechanism is adopted to capture the internal connections of interactions; each technique is assigned 4 independent DNNs with different configurations, resulting in 16 basic learners, and finally combined by deep neural network. To further evaluate the prediction performance of EnAmDNN, we apply it to 5 independent data sets, where improvements of various degrees can be observed for indicators AUC, ACC, Recall, Precision, F1, from which it can be concluded that EnAmDNN learns better than previous approaches from different DNNs and representations.

## Data Availability Statement

Alzheimer disease data was downloaded from the IntAct database (https://www.ebi.ac.uk/intact/query/annot:%22dataset:alzheimers%22?conversationContext=6) under search term annot: “dataset:alzheimers.” Cardiac data was downloaded from the IntAct database (https://www.ebi.ac.uk/intact/query/annot:%22dataset:cardiac%22?conversationContext=7) under search term annot: ‘dataset:cardiac.” Diabetes data was downloaded from the IntAct database (https://www.ebi.ac.uk/intact/query/annot:%22dataset:diabetes%22?conversationContext=8) under search term annot: “dataset:diabetes.” Parkinson's disease data was downloaded from the IntAct database (https://www.ebi.ac.uk/intact/query/annot:%22dataset:parkinsons%22?conversationContext=9) under search term annot: “dataset:parkinsons.” Cancer disease data was downloaded from the IntAct database (https://www.ebi.ac.uk/intact/query/annot:%22dataset:cancer%22?conversationContext=b) under search term annot: “dataset:cancer.” Protein sequence information were downloaded from the Uniprot database (https://www.uniprot.org/downloads).

## Author Contributions

Conceived and designed the experiments, performed the experiments, and contributed reagents, materials, analysis tools: FL. Algorithm design and analysis and analyzed the data: FZ and FL. Wrote the paper: FL, FZ, XL, and QL.

## Conflict of Interest

The authors declare that the research was conducted in the absence of any commercial or financial relationships that could be construed as a potential conflict of interest.
